# Discrimination and Cognition in Three Community-Based Cohorts: Think PHRESH, COBRA, and Heart-SCORE

**DOI:** 10.1093/geroni/igaf122.744

**Published:** 2025-12-31

**Authors:** Erica Fan, Sarah Royse, Tanisha Hill-Jarrett, Wendy Troxel, Tamara Dubowitz, Ann Cohen, Andrea Rosso

**Affiliations:** University of Pittsburgh School of Public Health, Pittsburgh, Pennsylvania, United States; University of Pittsburgh, Pittsburgh, Pennsylvania, United States; University of California Memory and Aging Center, San Francisco, California, United States; RAND, Pittsburgh, Pennsylvania, United States; University of Pittsburgh School of Public Health, Pittsburgh, Pennsylvania, United States; University of Pittsburgh, Pittsburgh, Pennsylvania, United States; University of Pittsburgh, Pittsburgh, Pennsylvania, United States

## Abstract

Previous work indicates an association between exposure to discrimination and poorer cognition in older adults; however, structural and demographic factors may affect the strength and direction of this association. Such factors differentially impact study enrollment and thus, without a clear understanding of how study design impacts these factors, inference from population studies may be limited. As such, in three cohort studies of cognitive health, we aimed to (1) examine differences in sample characteristics, (2) assess the association between discrimination and cognition, and (3) test the modifying effect of racialized group and study type. Of these community-based cohorts (n = 402), two included neuroimaging and all included older Black adults. Discrimination was measured using 5 items from the Everyday Discrimination Scale. Cognition was assessed using the Boston Naming test, Trails A/B, FAS fluency, and animal naming. Participants enrolled in imaging studies were more educated, reported lower perceived discrimination, and performed better on cognitive testing. We found that in the full cohort, discrimination was significantly associated with worse performance on total FAS fluency and animal naming (β=-0.26, p = 0.04; β=-0.13, p = 0.03) in linear regression models adjusted for age, sex, and education. The association between discrimination and total FAS fluency differed by racialized group (p = 0.005), where discrimination was not associated with cognition in Black participants across studies, but was associated with cognition in White participants. This suggests that the associations between discrimination and cognition may vary by racialized group; however, additional work and more sensitive measures may be needed to understand these complex, multifactorial relationships.

